# Supplementary material for: Content-rich biological network constructed by mining PubMed abstracts
Source: BMC Bioinformatics. 2004 Oct 8;5:147. doi: 10.1186/1471-2105-5-147 (PMC528731; doi:10.1186/1471-2105-5-147)
Supplement: Additional File 2 — The original results of the above study (non-essential files are deleted to keep the file size under the limit set by BMC bioinformatics). [file 1471-2105-5-147-S2.bz2 › chilibotAdditionalFile2/dip40/644ID8642594E11031/html/F_G.html]

 


 **F** and **G** 
  
Found 14343 abstracts in PubMed, retrieved 40.  
 

 What does Google say? 
 PDF only 
| .edu only 

---

**Interactive relationship** (e.g. stimulation, inhibition, etc)

**Neutral relationship**- Mice died from GVHD in group  **G**  were significantly more than those in group E and group  **F**  p <.  Ref: 12864951 Zhonghua Xue Ye Xue Za Zhi, 2003
- compared with 14 C FIAU, 18  **F**  FHBG led to as high %ID  **g**  in HSV1 tk expressing hepatocytes and to significantly greater %ID  **g**  in C6 stb sr39tk xenografts and HSV1 sr39tk expressing hepatocytes.  Ref: 12856156 Eur J Nucl Med Mol Imaging< MedlineTA>Eur J Nucl Med Mol Imaging, 2003
- Group  **F**  received plain bupivacaine 4 mg with 25 micro  **g**  of fentanyl and sterile water to a total of 1.5 ml, and Group B received only 0.5% plain bupivacaine 7.5 mg for spinal anaesthesia.  Ref: 12846615 Anaesthesia, 2003
- The  **F**  tra region encodes eight of the 10 highly conserved core gene products of T4SS including TraA  **F**  pilin, the TraB  **F** , K  **F**  secretin like, V  **F**  lipoprotein and TraC  **F**  NTPase, E  **F** , L  **F**  and TraG  **F**  N terminal region which correspond to TrbC P, I P,  **G**  P, H P, E P, J P, D P and TrbL P, respectively, of the P type T4SS exemplified by the IncP plasmid RP4.  Ref: 12855161 FEMS Microbiol Lett, 2003
- 5 FU concentrations in blood and tumor from blood concentration data were 435 in Group B, 2651 in Group C, 343 in Group E and 1538 in Group  **F** , while mean AUC 0 12 ng  **g** .h estimated from tumor tissue concentration data were 345 in Group B, 3548 in Group C, 324 in Group E and 2020 in Group  **F** .  Ref: 12853891 Anticancer Drugs, 2003

**Non-interactive relationship** (e.g. studied together, co-existance, homology, etc.)

- Compared with traditional heating, central heating significantly increases the risk of exposure to Der  **f**  1 levels > ; 2 μ **g**   **g**  of dust odds ratio, 7.35.  Ref: 12859558 AllergyAllergy, 2003
- CONCLUSIONS No differences were detected between patients treated with intra articular injections of Hylan  **G**   **F**  20 and those treated with the corticosteroid with respect to pain relief or function at six months of follow up.  Ref: 12851342 J Bone Joint Surg Am, 2003
- The patients treated with Hylan  **G**   **F**  20 received one course of three weekly injections.  Ref: 12851342 J Bone Joint Surg Am, 2003
- 0.05  **G**   **F**  compared with pigs fed the nonirradiated form.  Ref: 12854817 J Anim Sci, 2003
- RESULTS Both the group treated with the corticosteroid and the group treated with Hylan  **G**   **F**  20 demonstrated improvements from baseline WOMAC scores a median decrease from 55 to 40 points and from 54 to 44 points, respectively.  Ref: 12851342 J Bone Joint Surg Am, 2003
- Downstream, at the level of cAMP formation, all peptides were full agonists with the following rank order potency N OFQ 1 13 NH 2 >  **F**   **G**  O =  **F**   **G** .  Ref: 12853105 Neurosci Lett, 2003
- Over 2, and 16 wk, on alternate days, mice were treated with an intraperitoneal injection of either 1  **g** .kg 1.day 1 of human intact immunoglobulin or  **F**  ab 2 fragments of human immunoglobulin.  Ref: 12860569 Am J Physiol Heart Circ Physiol, 2003
- In GTPgamma 35 S binding  **F**   **G**  was a clear partial agonist with intrinsic activity E max stimulation factor, mean SEM, n=4 of 7.75 1.02 compared with N OFQ 1 13 NH 2 of 11.13 1.76.  Ref: 12853105 Neurosci Lett, 2003
- p = 0.06 or for those who received Hylan  **G**   **F**  20 median, 58 to 68 points.  Ref: 12851342 J Bone Joint Surg Am, 2003
- In an attempt to identify high potency NOP agonists for use in the brain we have compared the activity of a novel N OFQ analogue Phe 1 Psi CH 2 O Gly 2 N OFQ 1 13 NH 2  **F**   **G**  O with the existing Phe 1 Psi CH 2 NH Gly 2 N OFQ 1 13 NH 2  **F**   **G** .  Ref: 12853105 Neurosci Lett, 2003
- In the Mazatecan group the deletion frequency was 1.6%  **g** . **f** .  Ref: 12846056 Clin Exp Rheumatol< MedlineTA>Clin Exp Rheumatol, 2003
- Finally, pigs fed irradiated corn, whey, fishmeal, soybean oil, microingredients, or if all ingredients or the whole diet were irradiated, had similar ADG, ADFI, and  **G**   **F**  P > 0.12 to control pigs.  Ref: 12854817 J Anim Sci, 2003
- Molecular cloning identified six causative genes FANCA, C, D2, E,  **F** , and  **G**  encoding a multiprotein complex whose precise biochemical function remains elusive.  Ref: 12861027 Mol Cell BiolMol Cell Biol, 2003
- 14 C FIAU led to significantly higher %ID  **g**  in C6 stb tk xenografts than 18  **F**  FHBG P< ; 0.05.  Ref: 12856156 Eur J Nucl Med Mol Imaging< MedlineTA>Eur J Nucl Med Mol Imaging, 2003
- 0.01, had similar ADFI 2.40 0.03 vs 2.35 0.02 kg d, as fed basis and gain feed  **G**   **F** .  Ref: 12854801 J Anim Sci, 2003
- Since Phi values calculated from molecular dynamics simulations are the same as those determined experimentally, we can, with confidence, use the molecular dynamics simulations to analyse the structure of the transition state in detail, and are able to show loss of interactions between the A and  **G**  strands with associated A B and E  **F**  loops in the transition state.  Ref: 12850153 J Mol Biol, 2003
- In 3 H N OFQ binding and GTPgamma 35 S binding the rank order affinity and potency was N OFQ 1 13 NH 2 >  **F**   **G**  O >  **F**   **G** .  Ref: 12853105 Neurosci Lett, 2003
- The LD 50 was found at 1.6 μ **g**   **g**  95% fiducial limit  **f** .l. 0.7 to 2.2, the LD 80 at 2.7 μ **g**   **g**  95%  **f** .l.  Ref: 12851102 Toxicol Pathol< MedlineTA>Toxicol Pathol, 2003
- Group A received no treament, group B received TBI only, the rest groups were irradiated 9 Gy by 60 Co and transfused 10 5 CD 34 cells or mixed with 10 7 E, 10 8  **F** , 1.5 x 10 8  **G**  of CD 3 cells respectively.  Ref: 12864951 Zhonghua Xue Ye Xue Za Zhi, 2003
- The enhanced potency and intrinsic activity of the novel  **F**   **G**  O modification makes this an interesting peptide for further in vivo analysis.  Ref: 12853105 Neurosci Lett, 2003
- The efficacy of  **F**   **G**  O 10.17 1.88 approached that of the full agonist N OFQ 1 13 NH 2.  Ref: 12853105 Neurosci Lett, 2003
- 0.001, less  **G**   **F**  313 4 vs 341 3.  Ref: 12854801 J Anim Sci, 2003
- We report here the generation of a soluble form of TXAS by deletion of the amino terminal membrane anchoring domain and replacement of the helix  **F**  and  **F**   **G**  loop region with the corresponding region of the structurally characterized microsomal P450 2C5.  Ref: 12859980 Arch Biochem Biophys, 2003
- 1.9 to 4.0 and the LD 90 at 3.6 μ **g**   **g**  95%  **f** .l.  Ref: 12851102 Toxicol Pathol< MedlineTA>Toxicol Pathol, 2003
- The scores on the visual analog scale improved for patients receiving Hylan  **G**   **F**  20 median, 70 to 52 mm.  Ref: 12851342 J Bone Joint Surg Am, 2003
- In addition,  **G**   **F**  was improved P <.  Ref: 12854817 J Anim Sci, 2003
- Pigs fed the diet containing irradiated animal plasma had improved feed efficiency  **G**   **F** .  Ref: 12854817 J Anim Sci, 2003
- We tested the hypothesis that there are no significant differences between Hylan  **G**   **F**  20 Synvisc and the corticosteroid betamethasone sodium phosphate betamethasone acetate Celestone Soluspan in terms of pain relief or improvement in function, as determined by validated scoring instruments.  Ref: 12851342 J Bone Joint Surg Am, 2003
- Zoccali C, Mallamaci  **F** , Tripepi  **G** , Cutrupi S, Parlongo S, Malatino LS, Bonanno  **G** , Rapisarda  **F** , Fatuzzo P, Seminara  **G** , Stancanelli B, Nicocia  **G** , Buemi M Institute of Biomedicine, Clinical Epidemiology and Pathophysiology of Renal Diseases and Hypertension, Reggio Cal.  Ref: 12859694 J Intern MedJ Intern Med, 2003
- METHODS One hundred patients with knee osteoarthritis were randomized to receive intra articular injection of either Hylan  **G**   **F**  20 or the corticosteroid, and they were followed for six months.  Ref: 12851342 J Bone Joint Surg Am, 2003
- Tumor weights  **g** , mean SD were 0.54 0.15 in Group A, 0.52 0.17 in Group B, 0.34 0.05 in Group C, 0.46 0.12 in Group D, 0.34 0.07 in Group E and 0.16 0.03 in Group  **F** .  Ref: 12853891 Anticancer Drugs, 2003
- Given the recognized role of proinflammatory cytokines in septic shock and of extracellular  **F**  and  **G**  actin in the pathophysiology of multiple organ dysfunction, we have investigated the role of Tbeta 4 in sepsis.  Ref: 12860178 Int ImmunopharmacolInt Immunopharmacol, 2003
- Since three of these genera Finegoldia, Micromonas and Peptostreptococcus contain only a single species,  **F** . magna, M. micros and P. anaerobius, respectively, these organisms were identified to the species level directly by using the multiplex PCR  **G** .  Ref: 12855723 Microbiology< MedlineTA>Microbiology, 2003
